# Proteostasis of immune checkpoint receptors

**DOI:** 10.1042/BCJ20253299

**Published:** 2025-10-01

**Authors:** Pei Yee Tey, Sylvie Urbé, Michael J. Clague

**Affiliations:** 1 Biochemistry, Cell and Systems Biology, Institute of Systems, Molecular and Integrative Biology, University of Liverpool, Liverpool, U.K

**Keywords:** immune checkpoint, membrane trafficking, proteostasis, ubiquitin, Protein turnover

## Abstract

Immunotherapy relies on the targeting of immune checkpoint receptors and their respective ligands by specific antibodies that bind to the cell surface proteins. The pace of this highly successful clinical advancement has outstripped our cell biological understanding of these receptors. Here, we discuss what is known about their intracellular trafficking itineraries, which determine the bioavailability of these proteins for clinical targeting. Some of them are amongst the shortest-lived membrane proteins (CTLA-4), whilst others can be very stable (PD-L1). We highlight the ubiquitin system, which is key to determining their turnover, as it plays a key role in disposing of misfolded newly synthesised proteins via the ERAD pathway and generating a key signal for endosomal sorting towards lysosomes. In some cases, ubiquitylation can modulate the signalling function of the immune checkpoint receptor, as seen for LAG-3. Immune checkpoint proteins can evade lysosomal degradation by effective recycling to the plasma membrane using highly specialised factors, including CMTM6 (for PD-L1) and LRBA (for CTLA-4). Lastly, we consider how reprogramming the ubiquitin system emerges as an alternative modality in targeting immune checkpoint receptors.

## Introduction

**Figure 1:**
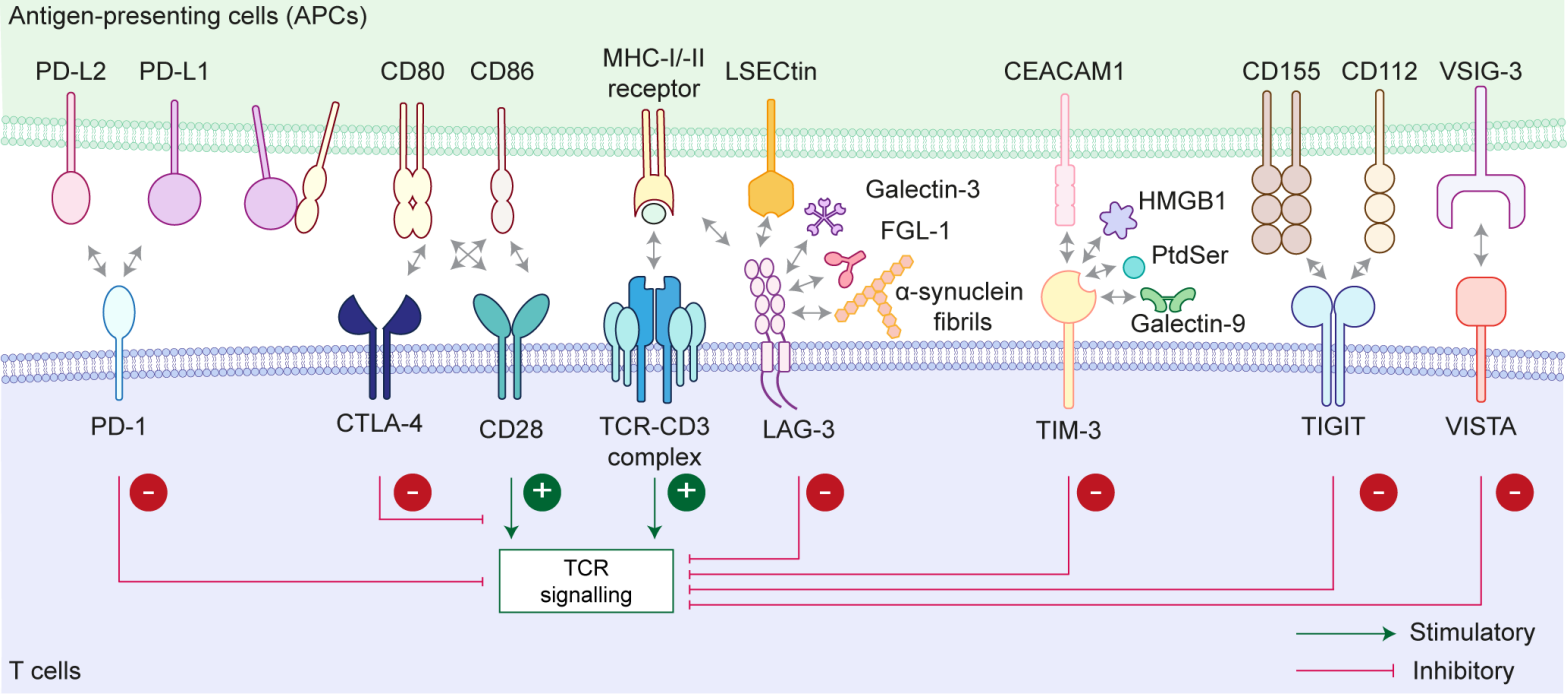
Immune checkpoint receptors and their ligands. The T-cell-mediated immune response relies on two distinct signals. It begins with antigen presentation by the major histocompatibility complex (MHC) molecules to the T cell receptor (TCR)–CD3 complex, which serves as the first activation signal. Antigen presentation via MHC receptors can be mediated by either MHC class I (MHC-I) or class II (MHC-II). MHC-I presents endogenous or viral antigens to CD8+ T cells, whereas MHC-II presents exogenous antigens, processed in the endolysosomal pathway, to CD4+ T cells. This process is accompanied by a co-stimulatory signal, where co-stimulatory receptors (e.g. CD28) interact with their ligands (e.g. CD80 and CD86) found on antigen-presenting cells (APCs). Note that several co-stimulatory receptors have been described, and only the chief co-stimulatory receptor, CD28, is depicted here. Immune checkpoint receptors, typically expressed on activated T cells, bind to their corresponding ligands on APCs to restrain stimulatory immune signalling. Most immune checkpoint receptors lack a clear molecular signature that defines their inhibitory roles. Generally, they can either initiate an inhibitory signalling cascade in immune cells (the best-characterised for PD-1) or displace co-stimulatory receptors for ligand binding (see CTLA-4). CD80 can be found in monomeric and dimeric forms, and monomeric CD80 can heterodimerise with PD-L1 in cis. This restricts dimeric CD80 interaction with CTLA-4 and PD-L1 binding to PD-1 but preserves CD80–CD28 binding, further adding another layer of complexity to how cell surface immune checkpoint receptors can modulate immune signalling output. PD-L1, programmed death ligand 1; PD-L2, programmed death ligand 2; PD-1, programmed cell death protein 1; CTLA-4, cytotoxic T lymphocyte-associated antigen 4; LAG-3, lymphocyte activation gene 3; TIM-3, T cell immunoglobulin and mucin domain-containing protein 3; TIGIT, T cell immunoreceptor with immunoglobulin and ITIM domains; VISTA, V-domain Ig suppressor of T cell activation; VSIG-3, V-set and Ig domain-containing 3; CEACAM1, carcinoembryonic antigen-related cell adhesion molecule 1; FGL1, fibrinogen-like protein 1; LSECtin, liver and lymph node sinusoidal endothelial cell C-type lectin; HMGB1, high-mobility group protein B1; PtdSer, phosphatidylserine.

The immune response to antigen presentation is limited by immune checkpoint receptors. Immune suppression relies on the interaction between these proteins and their ligands, typically on immune and target cells, respectively. Targeting these interactions has provided a great leap forward in oncology, particularly in tumour types bearing high mutational load. This is exemplified by therapeutic antibodies, which target programmed cell death receptor 1 (PD-1; *PDCD1*) on T cells or its cognate ligand programmed death ligand 1 (PD-L1; *CD274*) on antigen-presenting cells (APCs) [1]. Several immune checkpoint proteins have been described in different subsets of immune cells, including PD-1, cytotoxic T lymphocyte-associated antigen 4 (CTLA-4; *CTLA-4*), lymphocyte activation gene 3 (LAG-3; *LAG3*), T cell immunoglobulin and mucin domain-containing protein 3 (TIM-3; *HAVCR2*), T cell immunoreceptor with immunoglobulin and ITIM domains (TIGIT; *TIGIT*) and V-domain Ig suppressor of T cell activation (VISTA; *VSIR*) (Figure 1, Table 1). The functions of these receptors have been extensively characterised in activated CD8+ T cells, particularly in the settings of chronic infection and cancer, but it is important to note that they are also present on other lymphocyte populations and innate immune cells (Table 1), where they may modulate immune responses. Whilst these proteins are historically classed as receptors expressed by immune cells, in reality, some of them are also presented by cancer cells (Table 1), which may represent a strategic adaptation to avoid immune detection. Beyond cancer, dysregulated immune checkpoint receptor expression on T cells has been described in several autoimmune diseases [22–24].

**Table 1:** Immune checkpoint receptors and their reported ligands.

Receptor	Alternative name	Expressed by	Ligands	Refs.
PD-1	CD279	Activated T cells (CD4+ and CD8+), B cells, dendritic cells and cancer cells	PD-L1 and PD-L2	[2–5]
CTLA-4	CD152	Activated T cells (CD4+ and CD8+), FoxP3+ regulatory T cells and cancer cells	CD80 and CD86	[6–8]
LAG-3	CD223	Activated T cells (CD4+ and CD8+) and natural killer cells	MHC class II, galectin-3, fibrinogen-like protein 1 (FGL1), liver and lymph node sinusoidal enodthelial cell C-type lectin (LSECtin) and α-synuclein	[9–12]
TIM-3	HAVCR2	Activated T cells (CD4+ and CD8+), FoxP3+ regulatory T cells, mast cells, dendritic cells, monocytes, macrophages, natural killer cells, leukaemic stem cells and tumour-associated endothelium	Galectin-9, carcinoembryonic antigen-related cell adhesion molecule 1 (CEACAM1), high mobility group protein B1 (HMGB1) and phosphatidylserine	[13–18]
TIGIT	WUCAM, vstm3, VSIG9	Activated T cells (CD4+ and CD8+), FoxP3+ regulatory T cells and natural killer cells	CD155 and CD122	[19,20]
VISTA	PD-1H	Myeloid cells, naïve CD4+T cells and FoxP3+ regulatory T cells	V-Set and immunoglobulin domain containing 3 (VSIG-3)	[21]

A sustained T cell activation relies on the co-engagement of the T cell receptor (TCR)–CD3 complex and co-stimulatory receptor, such as CD28, with their respective ligands on the APCs (Figure 1). This interaction rapidly triggers a cascade of dynamic molecular events at the cytosolic interface in T cells, leading to the recruitment and activation of several tyrosine kinases, including the Src family kinases Lck and Fyn, as well as the Syk family kinase ZAP70 [25]. These signalling molecules assemble into an early TCR signalling site, known as the TCR microcluster, which then matures into a structured immunological synapse [25]. Unlike the TCR signalling pathway, which has distinct phosphorylation cascades and molecular events, many immune checkpoint receptors lack a direct molecular signature or specific biochemical readouts that clearly define their inhibitory function. Therefore, T cell functional assays, such as those measuring their proliferation, cytokine production and cytotoxicity, are often used to infer their inhibitory effects. The proposed mechanisms by which these immune checkpoints function have been extensively discussed elsewhere [19,26–30]. Broadly speaking, once expressed on the activated T cells, they can either (i) bind their respective ligands to instigate inhibitory signalling cascades in T cells, or (ii) compete with co-stimulatory receptors for ligand binding, thereby tuning down the immune responses. The distinct and co-ordinated expression patterns of the co-stimulatory and inhibitory immune receptors on the cell surface support a well-regulated immune response.

The premise behind immuno-oncology lies in the recognition that immune checkpoint receptors restrict the function of T cells that infiltrate tumours. This class of receptors must be functional and available at the cell surface to engage their respective ligands on APCs for eliciting their inhibitory functions. This also renders them accessible to neutralising antibodies, which disrupt receptor–ligand interaction. By liberating the constraint imposed by immune checkpoints, it is possible to revitalise T cell-mediated anti-tumour activities. This approach has transformed oncology practice, with ten immune checkpoint inhibitors approved for clinical use (targeting the PD-1/PD-L1 axis, CTLA-4 and LAG-3) and many more antagonists being evaluated under clinical trials [1,31].

The current suite of immune checkpoint inhibitors recognises cell surface-exposed proteins. However, it should be emphasised that these proteins are dynamically distributed throughout cellular compartments, and their intracellular trafficking itinerary and turnover will regulate their availability. It follows then that understanding factors which govern their turnover or surface expression may open up new biological targets for immune checkpoint regulation by small molecules. Given such prominence of immune checkpoint proteins in the therapeutic landscape, we highlight a deficit in our comprehension of their cell biology, particularly in relation to their turnover and intracellular sorting. Our review will begin by outlining the molecular machinery known to govern receptor trafficking in cells, with a foregrounding of reversible ubiquitylation as a key sorting signal. We will then discuss our current knowledge related to this class of receptors, focusing specifically on the turnover and membrane trafficking pathways of selected immune checkpoint receptors and their ligands.

## A brief overview of receptor trafficking: focus on reversible ubiquitylation

The ubiquitylation status of a protein reflects the overall activity of the ubiquitin conjugation cascade, which is mediated sequentially by the ubiquitin E1, E2 and E3 enzymes and can be counterbalanced by deubiquitylases (DUBs). It is the E3 ligases, of which there are more than 600, that recognise specific substrates for ubiquitylation [32]. They can be divided into four families according to their mechanism of action: homologous to the E6AP carboxyl terminus (HECT), really interesting new gene (RING), RING-between-RING (RBR) and U-Box. Ubiquitylation directs proteins for degradation via either the proteasomal or lysosomal pathways, wherein lysosomes are reached through endocytosis or autophagy pathways [33]. Proteasomal substrates commonly arise from the cytoplasm or the endoplasmic reticulum-associated protein degradation (ERAD) pathway. Ubiquitin is typically covalently attached to the lysine (Lys) residues on substrates via an isopeptide linkage. The presence of seven internal Lys residues alongside the N-terminal methionine (Met) allows for the formation of eight topologically distinct ubiquitin chain linkages (Met1, Lys6, Lys11, Lys27, Lys29, Lys33, Lys48 and Lys63), which provide the foundational layer of the ubiquitin code [34,35]. Lys48 and Lys63 are the most abundant chain types in most studied cell lines at interphase and have been broadly assigned to directing proteasomal and lysosomal degradation, respectively [36]. Besides its canonical function in mediating degradation, ubiquitylation has emerging non-proteolytic roles [37]. Removal or trimming of the polyubiquitin chains by DUBs is crucial to recycling ubiquitin or affecting its signal. DUBs fall into seven classes according to their catalytic domains: USP, OTU, JAMM/MPN, Josephin, UCH, MINDY and ZUP1. Among the ~100 DUBs, those from the JAMM/metalloprotease and OTU families often exhibit stringent ubiquitin chain linkage selectivity [38].

The endomembrane system comprises a set of highly organised membranous organelles that are spatially and functionally connected via vesicular flux. For about a third of newly synthesised proteins, their entry into the ER marks the beginning of their trafficking journey. In the ER, they undergo folding with the assistance of chaperones, formation of disulphide bonds and glycosylation, whilst being continuously monitored by the ER quality control pathways. Even with the enormous cellular investment dedicated to assisting protein folding, this process is inherently error-prone, and approximately 12–15% of newly synthesised proteins are co-translationally eliminated by the ubiquitin–proteasome system [39]. Proteins that fail to mature in the ER are targeted by the ERAD pathway, where they are first ubiquitylated and then extracted out of the ER for proteasomal degradation [40]. Alternatively, misfolded proteins that fail to engage with the ERAD machinery can be shuttled via vesicles to the lysosomes through the ER-to-lysosome-associated degradation pathway [41]. Fully folded and assembled proteins will be incorporated into COPII-coated vesicles destined for the Golgi apparatus. As they pass through the Golgi, proteins are further glycosylated before being sorted at the trans-Golgi network (TGN) to multiple destinations, including the plasma membrane [42].

Cell surface receptors that enter the endocytic pathway are typically encapsulated in clathrin-coated vesicles (CCVs) for delivery to the tubulovesicular compartments known as sorting or early endosomes [43]. Here, sorting occurs due to endomembrane partitioning into distinct microdomains on their cytosolic face, concentrated in the endosomal sorting complex required for transport (ESCRT) or the recycling machinery, respectively [44,45]. This active sorting sits in equilibrium between degradative and recycling systems, which ultimately determines the localisation and destiny of endocytosed receptors. One of the key determinants is the ubiquitylation status of receptors. This was initially discovered in yeast and shortly thereafter shown for the ubiquitin E3 ligase Cbl-mediated degradation of activated EGF receptors [46,47]. Notably, fusion of a ubiquitin molecule to transferrin receptors can reroute them from their usual recycling pathway to lysosomal degradation [48].

Ubiquitylated receptors are captured by the ESCRT machinery at the limiting membrane of the endosomes. ESCRT-0 (HRS and STAM) offers an initial five ubiquitin-binding sites, and additional sites are provided by ESCRT-I (TSG101, MVB12 and UBAP1) and -II (EAP45) components [49]. This mediates their inclusion into inwardly budding membranes to form the intraluminal vesicles (ILVs) of the maturing endosomes, otherwise termed multivesicular bodies (MVBs) [49–54]. The late-acting ESCRT-III proteins do not encode ubiquitin-binding domains; instead, they drive membrane scission to complete the sequestration of receptors. Mature MVBs fuse either with the lysosome, where receptors will be degraded, or with the plasma membrane to release the ILVs as exosomes [55,56]. Conversely, the recycling machinery recognises motifs on specific receptors and actively directs them away from the lysosomal degradative pathway back to the plasma membrane [57,58].

The strict requirement for ubiquitylation lies in its ability to direct receptor sorting at the endosome [59–61]. Whilst it may be dispensable for receptor internalisation, modification of receptors by polyubiquitin, particularly those linked via Lys63 linkage, is needed for efficient receptor sorting into the MVBs [62–64]. Ubiquitin binding is co-operative, and it is not surprising that the length of ubiquitin chains can dictate the fate of receptors. A prominent example of such is major histocompatibility complex-II (MHC-II), which exhibits a distinct subcellular localisation in resting dendritic and B cells. Immature dendritic cells sequester MHC-II in the MVB and lysosomes, whereas B cells retain MHC-II at the cell surface. This difference arises from a shorter MHC-II-conjugated ubiquitin chain in B cells (2–3 ubiquitin conjugates in B cells compared with 4–6 ubiquitin conjugates in immature dendritic cells) [65].

The compartmentalisation of the ubiquitin system within cells allows for the generation or termination of localised ubiquitylation events along the biosynthetic and endocytic pathways [32,66]. This is exemplified by the ER-resident E3 ligase, HRD1, which prominently ubiquitylates misfolded or unassembled proteins, such as the TCR-α chain, for destruction by the ERAD pathway [67]. The DUB USP19 is situated on the ER membrane and can reverse the commitment to the ERAD pathway [68]. Along the endocytic pathway, the ubiquitin E3 ligase, c-Cbl, is recruited to activated receptor tyrosine kinases (RTKs) at endosomes, leading to receptor ubiquitylation and sequestration into the MVB for lysosomal down-regulation [69]. Inclusion into MVBs spatially separates the RTKs away from the cytosol to terminate their signalling events. Two endosome-associated DUBs, AMSH (STAMBP) and USP8 (UBPY), interact with members of the ESCRT machinery but exert opposite effects on the degradation kinetics of RTKs [51,70–76]. AMSH, which displays high stringency for the Lys63-linked polyubiquitin chain, has been proposed to rescue ubiquitylated receptors from a degradative fate by reversing their ubiquitylation [51,75]. By contrast, USP8 is crucial for effective receptor degradation, likely due to its activity against most polyubiquitin chain linkages and its key role in stabilising the ESCRT-associated components, such as HRS and STAM [51,71,77,78]. c-Cbl, AMSH and USP8 provide notable examples of how coupling enzymatic activity to its endosomal localisation can influence the fate of internalised receptors.

## Turnover of the immune checkpoint receptors

Protein turnover refers to the net balance between protein synthesis and degradation, which defines the stability of a protein. At steady state, the turnover rate and protein half-life (t_1/2_) are equivalent and can be a significant determinant of protein abundance [79,80]. Modern mass spectrometry approaches have afforded global views of cellular protein stability, following isotopic labelling with amino acids or simply by measuring decay in abundance following translational arrest with cycloheximide [81]. In many tissue culture cell types, only ~5% of the proteome has a half-life of less than 8 h [82]. A corresponding dataset acquired from resting and activated T cells, unfortunately, did not register the checkpoint receptors in their turnover experiments [83].

Receptors involved in initiating immune responses can display different half-lives depending on the activation status of the cells or cell types. The TCR-CD3 complex is made up of a heterodimeric TCR (TCR-α and TCR-β chains) and a multimeric CD3 complex (CD3-γ, CD3-δ and CD3-ε). Unpaired TCR-α chain and CD3-ε are targeted for proteasomal degradation via the ERAD pathway (t_1/2_ ≈ 1 h) [67,84]. When correctly assembled, the TCR–CD3 complex is fairly long-lived in resting T cells, maintained by continuous internalisation and fast recycling back to the plasma membrane [85]. Nevertheless, it undergoes ubiquitylation after antigen stimulation, leading to its lysosomal down-regulation to protect T cells from overstimulation [85–87]. This process was first attributed to the Cbl family E3 ligases, c-Cbl and Cbl-b, and was later shown to also involve another E3 ligase, GRAIL [88,89]. Similarly, whilst the co-stimulatory receptor, CD226, is typically stable on the surface of resting T cells and natural killer cells, it undergoes Cbl-b-mediated ubiquitylation and degradation upon engagement with its ligand, CD155 [90]. In contrast with this dynamic receptor regulation, the primary co-stimulatory receptor, CD28, is constitutively expressed by T cells and remains stable regardless of their activation status [91].

This principle of dynamic receptor regulation extends beyond immune signalling receptors to antigen-presenting receptors. MHC class I and II receptors are loaded with antigens within the ER and endosomal compartments, respectively. They are themselves subject to intracellular trafficking and ubiquitylation-dependent control [92,93]. This is critical for immune activation but can also be exploited by pathogens and tumours. In immature dendritic cells, MHC-II is ubiquitylated by the E3 ligase MARCH1 for lysosomal degradation (t_1/2_ ≈ 4 h), but its ubiquitylation diminishes as dendritic cells mature, leading to its stabilisation at the plasma membrane (t_1/2_ > 8 h) [94–97]. This switch ensures that only mature dendritic cells are competent to sustain antigen presentation to CD4+T cells, thus avoiding premature immune activation. Peptide-loaded MHC-I is a stable heterotrimer (t_1/2_ ≈ 8–30 h) composed of heavy chain (HC), β2-microglobulin (β2m) and a peptide antigen derived from either self or viral proteins [98]. In the absence of appropriate complex assembly, nascent MHC-I HC is targeted for ERAD degradation by the ubiquitin E3 ligase HRD1 (t_1/2_ ≈ 80 mins) [99]. During infection, certain viruses exploit the host machinery to destabilise MHC-I for blocking antigen presentation. For example, human cytomegalovirus (HCMV) encodes US2 and US11, which mediate MHC-I HC ubiquitylation via the host ER-resident E3 ligases, RNF139 (TRC8) and TMEM129, respectively (t_1/2_ < 5 mins) [100–103]. Similarly, Kaposi’s sarcoma-associated herpesvirus (KSHV) encodes the viral E3 ligases K3 (MIR1) and K5 (MIR2) to drive lysosomal degradation of MHC-I (t_1/2_ < 1 h) [104,105]. Analogous to viral immune evasion, many tumours down-regulate MHC-I to escape cytotoxic CD8+ T cell responses. Whilst several mechanisms contribute to reduced MHC-I expression in tumours, a recent study showed that this also involves recruitment of the ubiquitin E3 ligase WWP2 by the SUSD6/TMEM127 complex to promote MHC-I ubiquitylation and lysosomal degradation [106,107].

For immune checkpoint receptors, our understanding of their stability, especially the impacts of ligand binding on their turnover rates, is relatively sparse. In Table 2, we have collated the known half-lives of these receptors and their ligands. It must be noted that most of these studies were performed in host tissue culture cell lines, where the rates of protein synthesis, expression patterns of E3 ligases and DUBs, and proteasomal activity are likely different from those in primary T cells and tumours, which are influenced by the tissue microenvironment and physiological cues [124]. Therefore, the reported half-lives should be interpreted as indicative rather than absolute values.

**Table 2:** Reported half-life of immune checkpoint receptors and their ligands.

Receptor/ligand	Reported half-life (t_1/2_)	Refs.
PD-1	~72 mins in RAW264.7 cells ~6 h in MOLT-4 and THP-1 cells < 6 h in HEK293T cells (exogenous PD-1) > 6 h in U2OS (endogenous PD-1) and HEK293T cells (exogenous PD-1) > 6 h in Jurkat cells	[108] [109] [110] [111] [112]
PD-L1	< 40 mins in LOVO cells < 3 h in HEK293T cells (exogenous PD-L1) < 8 h in HeLa cells released from nocodazole > 6 h in MDA-MB231 (endogenous PD-L1) and HEK293T cells (exogenous PD-L1) > 8 h in RPE1 and U2OS cells > 12 h in A549 and NCI-H358 cells	[113] [114] [115] [116,117] [82] [118,119]
PD-L2	> 8 h in SCC-15 cells > 8 h in RPE1 cells	[120] [82]
CTLA-4	< 1 h in A2058 and NCI-H520 cells ~2 h in activated transgenic T cells	[121] [91]
LAG-3	< 2 h in resting and activated Jurkat cells (exogenous LAG-3)	[122,123]

Our working assumption is that most of the mature receptors are degraded after sorting to the lysosome. There is little evidence for extraction from endomembranes for proteasomal degradation except at the ER, where misfolded proteins will enter the ERAD pathway. Lysosomal degradation may be confirmed by rescue with v-ATPase inhibitors such as concanamycin [81]. We are familiar with instances where the lysosomal degradation of membrane receptors (e.g. Met) has been misinterpreted following rescue by proteasomal inhibitors [125]. This has been shown to reflect a depletion of the free ubiquitin pool required for receptor sorting rather than a direct block to receptor degradation itself [126]. In line with this, we advise caution in interpreting proteasome-dependent protein expression to measure receptor stability without corresponding data on lysosomal inhibition.

PD-1 and PD-L1 are reportedly degraded by both the proteasomal and lysosomal pathways, but some of these studies may be subject to the caveats described above [108,110,113–115,127–129]. In many cancer cell lines, PD-L1 is a highly stable protein, which may facilitate cancer immune evasion by sustaining inhibitory signalling to T cells (Table 2). Unlike their mature counterparts, immature PD-1 and PD-L1 can be degraded rapidly (t_1/2_ < 2 h) in a proteasome-dependent manner via the ERAD pathway [111,116,130,131]. These fast turnover kinetics were established using cycloheximide treatment and glycosylation-deficient mutants, for which the key asparagine residues are mutated to glutamine [111,116,130,131]. It is currently unknown what proportion of nascent PD-1 and PD-L1 proteins are naturally eliminated by the ERAD pathway before their maturation in the ER, but global studies of degradation of newly synthesised proteins have suggested it can be up to 30% [132].

## Trafficking itinerary of immune checkpoint receptors

The turnover of membrane receptors is inherently connected to their intracellular sorting. Endocytosis and lysosomal degradation provide means to terminate signals by controlling receptor distribution and turnover [49,50]. Studies on the membrane trafficking pathways governing PD-1 and its ligands PD-L1 and PD-L2, as well as CTLA-4, LAG-3 and TIM-3, have so far provided rough sketches of each itinerary. In contrast, the trafficking routes for other immune checkpoint receptors like TIGIT and VISTA represent uncharted territory. A crucial step towards a fuller understanding lies in delineating their trafficking routes and establishing how ubiquitin-modifying enzymes influence each of their trafficking steps.

### PD-L1

PD-L1 has drawn a lot of attention due to the remarkable success of inhibitors targeting the PD-1/PD-L1 axis. Blocking this pathway effectively restores T cell-mediated anti-tumour immunity because many tumours exploit PD-L1 up-regulation, often driven by oncogenic signalling, inflammatory cytokines and high tumour mutational burden [133]. Consequently, PD-L1 has become one of the most intensively studied targets in immunotherapy, and as such, its cell biology is comparatively more mature than the other immune checkpoint receptors. PD-L1 is not a classical signalling receptor, but its binding to PD-1 is crucial to activate PD-1 inhibitory signals in T cells (see below). PD-L1 and PD-1 are monomeric proteins that normally interact in trans between adjacent cells, but they can also interact in cis when PD-1 is expressed by APCs [134]. This latter mode of interaction can be more prevalent due to the spatial proximity of the molecules on the same membrane, blocking PD-L1/PD-1 interaction in trans and limiting PD-1 inhibitory signal in T cells [134]. Besides interacting with PD-1, PD-L1 can also bind to monomeric CD80 on the same cells (Figure 1). This disrupts CD80/CTLA-4 binding and PD-L1/PD-1 interaction in trans, thus limiting PD-1 and CTLA-4-mediated immune inhibition, but preserves CD80/CD28 interaction [135,136]. These multiple interactions add further complexity to how surface PD-L1 can modulate immune responses (Figure 1).

#### Biosynthesis 

**Figure 2:**
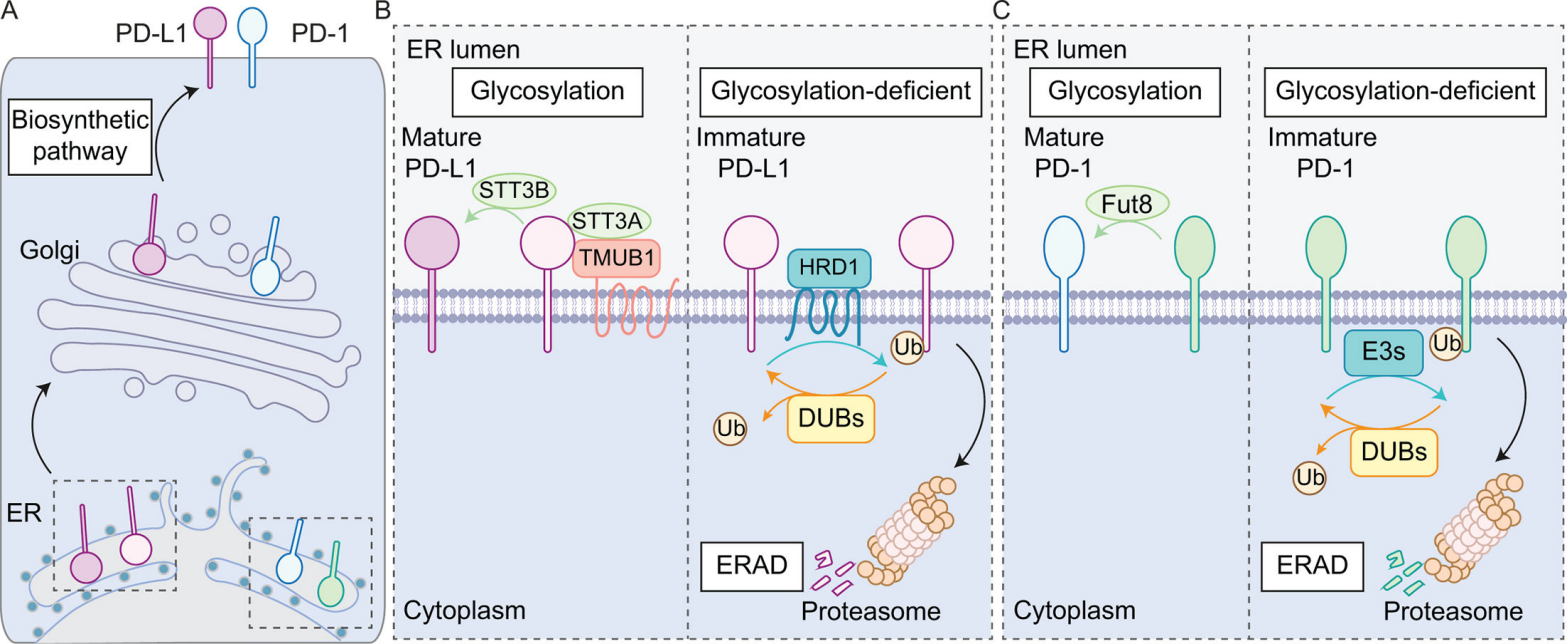
Glycosylation protects PD-L1 and PD-1 from proteasomal degradation via ERAD. **A.** Glycosylation ensures the maturation of PD-L1 and PD-1 for surface presentation. **B.** PD-L1 glycosylation is mediated by the N-glycosyltransferases STT3A and STT3B, wherein STT3A is recruited by the ER-associated TMUB1. Incompletely glycosylated PD-L1 is ubiquitylated by HRD1 for destruction in the proteasome via the ERAD pathway. **C.** PD-1 is glycosylated by Fut8, without which it is ubiquitylated for proteasomal degradation via ERAD. TMUB1, transmembrane and ubiquitin-like domain-containing protein 1.

The ER-associated N-glycosyltransferases, STT3A and STT3B, mediate PD-L1 N-glycosylation for maturation and transport to the plasma membrane [137]. Transmembrane and ubiquitin-like domain-containing protein 1 (TMUB1) facilitates the recruitment of STT3A to PD-L1 [138]. Depletion of N-glycosyltransferases or mutations in the primary N-glycosylation residues (Asn35, Asn192, Asn200 and Asn219) in PD-L1 lead to a downshift, on an SDS-PAGE gel, of glycosylated PD-L1 (~50 kDa) to a non-glycosylated form (~30 kDa), which is degraded by the ERAD pathway [116,137]. It is noteworthy that the presence of glycan moieties on PD-L1 has been shown to hinder PD-L1 detection by antibodies, leading to potential misinterpretation of PD-L1 expression by various experimental approaches [139]. Several regulatory pathways have been suggested to govern the ERAD degradation of PD-L1. For example, metformin-induced PD-L1 phosphorylation by AMP-activated protein kinase (AMPK) results in abnormal PD-L1 glycosylation and prevents PD-L1 ER-to-Golgi transport, leading to PD-L1 ubiquitylation by the ERAD-associated HRD1 ligase (Figure 2) [130]. The DUBs OTUB1, OTUB2 and USP22 have all been suggested to oppose the PD-L1 ERAD pathway by reversing its ubiquitylation [117,118,140].

#### Intracellular dynamics

**Figure 3:**
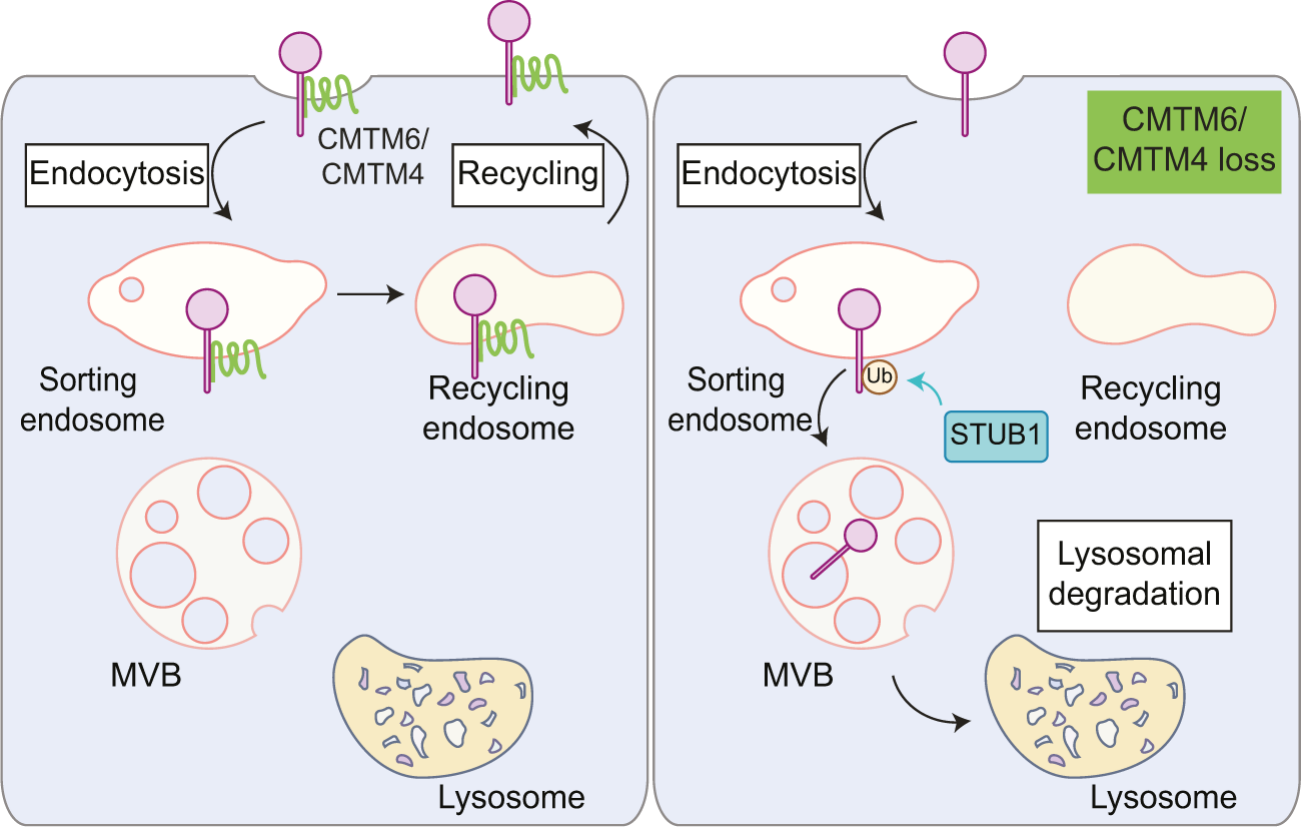
CMTM6 is the master regulator of PD-L1 recycling. Endocytosed PD-L1 constitutively binds CMTM6 and is recycled back to the plasma membrane. In the absence of CMTM6, internalised PD-L1 is ubiquitylated by STUB1 for lysosomal degradation. Homologous protein, CMTM4, can compensate for the loss of CMTM6 and recycle PD-L1 back to the plasma membrane in CMTM6-deficient cells. CMTM, CKLF-like MARVEL transmembrane domain containing.

Cell surface PD-L1 undergoes constitutive internalisation, after which it is rapidly deflected from the lysosomal pathway and recycled. The key factor is the tetraspanin protein CKLF-like MARVEL transmembrane domain containing 6 (CMTM6), a ubiquitous protein, predominantly expressed at the plasma membrane. CMTM6 was a unique outlier within two independent global loss-of-function screens for PD-L1 surface expression [127,141]. Dawson and colleagues used a genome-wide FACS-based CRISPR-Cas9 screen for surface PD-L1 expression in pancreatic cancer BxPC-3 cells, whilst Schumacher and colleagues conducted a similar genetic screen in IFN-γ-treated haploid HAP1 cells [127,141]. We and others have confirmed this robust effect of CMTM6 in maintaining PD-L1 stability in other cell lines [129,142]. Both groups elegantly showed that CMTM6 is not involved in PD-L1 egress from the biosynthetic pathway by analysing the maturation of newly synthesised PD-L1 [127,141]. Rather, CMTM6 recycles internalised PD-L1 back to the plasma membrane, whereas in its absence, PD-L1 undergoes lysosomal degradation (Figure 3) [127]. The ubiquitin E3 ligase, STUB1, is suggested to promote PD-L1 lysosomal degradation, as its depletion can compensate for the loss of PD-L1 in CMTM6-deficient cells [141]. In a modified genetic screen in CMTM6-deficient HAP1 cells, the homologous protein CMTM4, but not the other CMTM proteins, emerged as another PD-L1 regulator [141]. In CMTM6-deficient cells, CMTM4 can compensate for CMTM6 depletion by sustaining PD-L1 recycling to the cell surface [141]. It is surprising that our understanding of the biology of CMTM6 has not significantly advanced in the ensuing eight years, and it is still not clear whether this pathway involves established elements of recycling pathways such as Retromer, Retriever or Commander complexes [58]. As none of these genes were identified in the above screens, this may suggest some redundancy. Only a handful of high-confidence interacting proteins have been identified for CMTM6, and CMTM6 loss has little effect on the cell surface proteome beyond PD-L1 [127]. In IFN-γ-treated MDA-MB-231 breast cancer cells, its absence leads to the down-regulation of three proteins – cell adhesion molecule 1 (CADM1), scavenger receptor class F member 2 (SCARF2) and syntaxin binding protein 6 (STXBP6) – whilst up-regulating the expression of one, glutathione hydrolase 5 proenzyme (GGT5), but sequence alignment has not revealed any shared features among these proteins [127]. CMTM6 also interacts with another immune checkpoint receptor, LAG-3, but its depletion has no effect on LAG-3 expression [127]. Intriguingly, a recent total proteomic analysis of CMTM6-deficient cells revealed that the loss of CMTM6 down-regulates CD58, a ligand for the co-stimulatory receptor CD2 [143]. Together, these findings suggest that CMTM6 may act as a central regulator modulating the co-stimulatory and inhibitory immune pathways. Trafficking protein particle complex subunit 4 (TRAPPC4) was identified as a PD-L1 interactor in a mass spectrometry analysis of immunoprecipitated PD-L1 from a colorectal cancer cell line [128]. It is proposed to be a recycling adaptor for PD-L1 in conjunction with Rab11 GTPase, but whether this process involves CMTM6 remains unclear. PD-L1 recycling is favoured by palmitoylation of its cytoplasmic domain at the Cys272 residue by palmitoyltransferase DHHC3 [129]. PD-L1 palmitoylation does not appear to influence its interaction with CMTM6. Instead, it blocks PD-L1 ubiquitylation and ESCRT-mediated lysosomal degradation. Besides palmitoylation, alkylation of PD-L1 at Cys272, induced by cellular uptake of itaconate (a metabolic by-product of activated macrophages), also interferes with PD-L1 ubiquitylation and promotes its expression [144].

Endocytosed PD-L1 that is not recycled back to the plasma membrane has two principal potential fates: degradation in the lysosome or secretion via exosomes. Huntingtin-interacting protein 1-related (HIP1R), which contains a dileucine internalisation motif, is reported to interact with PD-L1 to mediate its lysosomal degradation [113]. This process is argued to be ubiquitin-independent, as the depletion of VPS28 (ESCRT-I) does not affect the levels of a GFP-fusion protein containing this HIP1R sorting motif. However, this fails to take into account the redundancy of ubiquitin-binding within this machinery [61,145]. Apart from HIP1R, the chaperone protein Hsc70 and a SEC16-interacting protein, TFG, are reported to mediate PD-L1 lysosomal degradation [142]. Hsc70 has been shown to compete with CMTM6 for PD-L1 binding, and its overexpression could prevent PD-L1 loss in CMTM6-deficient cells [142].

Once incorporated into the ILVs, PD-L1 can be released from cells via exosomes in an ALIX (PDCD6IP)-dependent manner [146]. ALIX depletion impairs PD-L1 trafficking through the endo-lysosomal pathway and down-regulates its packaging into exosomes, leading to an enhanced PD-L1 surface distribution. PD-L1 can also be shed from the cells following proteolytic cleavage by plasma membrane metalloproteases ADAM10 and ADAM17 [147]. This generates a soluble extracellular PD-L1 fragment (~37 kDa) and a residual PD-L1 fragment (~15 kDa), which is readily degraded in the lysosomes. Whilst PD-L1 exosomal release via ALIX correlates with low immunosuppression in the local tumour environment, the immunological consequences of PD-L1 shedding are currently unknown.

The homotypic fusion and vacuolar protein sorting (HOPS) and class C core vacuole/endosome tethering (CORVET) complexes are homologous tethering complexes critical to maintaining endo-lysosomal maturation and endosomal fusion [148]. A recent *in vivo* CRISPR-Cas9-focused screen used melanoma B16 cells, transduced with specific sgRNAs targeting these complexes, for transplantation into either immune-compromised or immune-competent mice [119]. This screen identified VPS11 and VPS18 as proteins whose loss sensitises tumours to immune elimination. In distinction to their canonical function, VPS11 and VPS18 are postulated to recycle PD-L1 back to the TGN in a Rab11 (recycling endosome) and VPS35 (retromer)-dependent manner [119]. It remains unclear how these spatially distinct endomembrane trafficking regulators act together to mediate PD-L1 recycling, but this process is suggested to refresh PD-L1 glycosylation for its continual plasma membrane delivery. A similar concept has been recently proposed for other plasma membrane glycoproteins, whereby low pH-dependent glycan removal is followed by retrograde transport to the Golgi to reset the glycan composition [149]. As for PD-L1, the specific enzymes and molecular cues involved in replenishing its glycan makeup have yet to be identified.

As of 2025, there are >300 studies on PD-L1 turnover and ubiquitylation with multiple DUBs and E3 ligases reported to affect its stability and ubiquitylation (Table 3). Many of these ubiquitin-modifying enzymes were identified as interactors by proteomic analysis, but there is little convergence between studies. For example, the JAMM DUB family member, CSN5, is proposed to stabilise PD-L1 in response to TNF-α stimulation by reversing its ubiquitylation [154]. It must be noted that this effect is likely indirect, as CSN5 is actually a deneddylase. Together with an inactive paralogue CSN6, it forms the catalytic core of the COP9 signalosome that removes the small ubiquitin-like molecule NEDD8 from the Cullin subunit of Cullin RING E3 ligases. This modification, neddylation, is essential for the ubiquitylation catalysed by these enzymes [165]. We do not intend to debate these findings in detail but rather highlight a lack of consensus in this area of research, in distinction to the highly robust results described for CMTM6 above. The application of specific small-molecule DUB inhibitors that have recently been developed will provide a simple orthologous approach for testing these claims. For example, highly specific inhibitors for USP9X and USP7 have been introduced lately, which could be used to clarify the current contradictory literature regarding the involvement of these two enzymes in the regulation of PD-L1 stability [117,140,156,158,166,167].

**Table 3:** Reported E3 ligases or DUBs regulating the immune checkpoint receptors and their ligands. E3 ligases or DUBs reported to govern the stability of immune checkpoint receptors (white) and their ligands (grey) are shown in blue and yellow, respectively.

Receptor/ligand	E3s or DUBs	Refs.
PD-1	MDM2	[111]
	KLHL22	[131]
	FBW7	[109]
	FBXO38	[110]
	c-Cbl	[108]
	TRIM21	[150]
	USP5	[112]
PD-L1	ß-TrCP	[116]
	HRD1	[130]
	RNF125	[151]
	ARIH1	[114]
	STUB1	[141]
	Cullin3-SPOP	[115]
	FBXO22	[152]
	MARCH8	[153]
	OTUB1	[117]
	OTUB2	[118]
	CSN5	[154]
	USP5	[155]
	USP7	[156]
	USP8	[157]
	USP9X	[158]
	USP21	[159]
	USP22	[140]
CTLA-4	TRAF6	[160]
	USP8	[121]
CD86	MARCH1	[161]
LAG-3	c-Cbl, Cbl-b	[123]
	LUBAC	[162]
	OTULIN	[162]
MHC-II	MARCH1	[94]
	MARCH8	[163]
TIM-3	HRD1	[164]

### PD-L2

The PD-L1 paralogue PD-L2 shows three-fold stronger binding affinity to PD-1, but its cell biology is not well developed [168]. This reflects that its expression was believed to be confined to professional APCs rather than tumour cells, in contrast with PD-L1. However, recent work has highlighted high levels of aberrant PD-L2 expression in various human cancers, including renal cell carcinoma, pancreatic cancer, head and neck squamous cell carcinoma and lung squamous cell carcinoma [169]. PD-L2 is glycosylated on Asn157, Asn163 and Asn189 by fucosyltransferase, Fut8 [120]. Glycosylation is proposed to prevent PD-L2 ubiquitylation and subsequent lysosomal degradation, which in turn can be blocked by inhibition of the v-ATPase [120]. The mechanism by which glycosylation-deficient PD-L2 is directed to the lysosomal pathway remains unclear, though we think it may indicate the involvement of a direct ER-to-lysosome-associated degradation pathway [41].

### PD-1

PD-1 has one of the best-characterised and reproducible molecular inhibitory signatures, mediated via its immunoreceptor tyrosine-based inhibitory motif (ITIM) and immunoreceptor tyrosine-based switch motif (ITSM) [170,171]. Ligand activation triggers PD-1 translocation to TCR microclusters, where it recruits the phosphatase SHP2 to dephosphorylate key signalling molecules within the TCR–CD3 complex and attenuates T cell signalling [28,172].

Similar to PD-L1, glycosylation protects newly synthesised PD-1 from misfolding and degradation, licensing its transport to the plasma membrane as a mature form (~50 kDa) [173,174]. A genome-wide CRISPR-Cas9 loss-of-function screen using a murine T cell hybridoma model reveals that the core fucosylation pathway is crucial in governing PD-1 surface delivery [173]. Specifically, genetic ablation or pharmacological inhibition of fucosyltransferase Fut8 promotes PD-1 degradation and down-regulates its surface levels (Figure 2) [173,174]. In addition to glycosylation-related genes, this screen also uncovered several components of the ubiquitin proteasome system that could potentially modulate PD-1 turnover, including USP7, TRIM23, FBXW14, FBXO28, FBXO47 and RNF43 [173]. Glycosylation-deficient PD-1 mutant is reported to be ubiquitylated by the ubiquitin E3 ligase MDM2 at its N-terminal Lys78 for degradation by the proteasome via the ERAD pathway [111]. It remains unclear how this ER-lumen-facing Lys residue can be reached by MDM2, a cytosolic and nuclear E3 ligase, as import is usually co-translational. MDM2 has also been reported to recruit N-glycanase, NGLY1, for PD-1 deglycosylation, but whether this happens in the biosynthetic or post-endocytic pathways remains to be assessed [111]. KLHL22, a Cullin 3 RING E3 ubiquitin ligase substrate receptor, whose expression is up-regulated upon T cell activation, is reported to ubiquitylate incompletely glycosylated PD-1 before its maturation [131].

PD-1 is internalised from the plasma membrane, but its post-endocytic fate is poorly understood. The transcription factor, thymocyte selection-associated high mobility group box protein (TOX), is reported to have an unconventional role in stabilising PD-1 in exhausted T cells infiltrating liver cancer [175]. Cytoplasmic TOX is proposed to shuttle PD-1 away from the lysosomal degradation pathway in favour of its recycling. When this process is disrupted, it accelerates PD-1 turnover and reduces its surface levels. FBXO38 was first reported to mediate Lys48-linked ubiquitylation of endocytosed PD-1 to promote its degradation [110]. This was claimed to be proteasomal, based on an increase in PD-1 surface levels following proteasomal inhibition by MG132. As discussed earlier, this may reflect a loss of the free ubiquitin pool required for lysosomal sorting, rather than a direct inhibition of proteasomal degradation. Additionally, a recent study challenges the role of FBXO38 in PD-1 regulation, reporting no evidence that FBXO38 influences PD-1 ubiquitylation, turnover and overall levels in T cells [176]. FBW7, TRIM21 and c-Cbl are also reported to target PD-1 for degradation, but it is unclear at which point during PD-1 trafficking these E3 ligases act [108,109,150].

### CTLA-4

**Figure 4:**
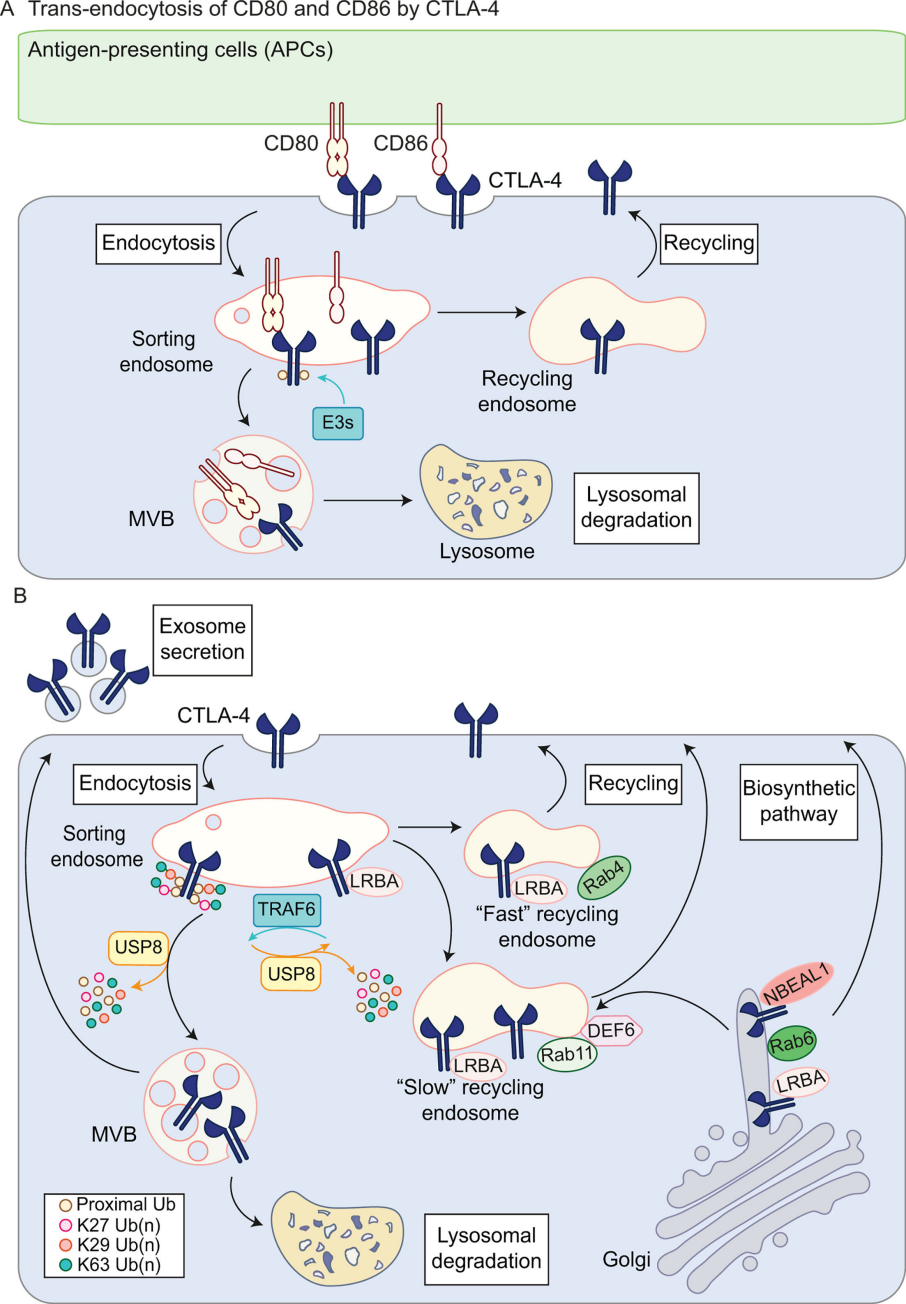
The dynamics of CTLA-4 sorting. **A.** Trans-endocytosis of CD80 and CD86 by CTLA-4. CTLA-4 internalises both of its ligands, CD80 and CD86, from the APCs. Low pH at the sorting endosome dissociates CTLA-4 from CD86, allowing its recycling back to the plasma membrane whilst CD86 is targeted for lysosomal degradation. CTLA-4 remains bound to CD80 and is ubiquitylated for inclusion in the MVB for lysosomal degradation. **B.** Clathrin-dependent CTLA-4 endocytosis is required for its ubiquitylation on the endosome and rapid lysosomal turnover. CTLA-4 is modified with a complex ubiquitin chain architecture comprising Lys63, Lys27 and Lys29 chains. TRAF6 is proposed to ubiquitylate CTLA-4, whilst endosomal USP8 removes ubiquitin from CTLA-4 before and after commitment to lysosomal degradation. Recycling of CTLA-4 to the plasma membrane is mediated by LRBA through ‘fast’ (Rab4-dependent) and ‘slow’ (Rab11-dependent) recycling pathways, the latter of which also involves DEF6 in T cells. In addition, LRBA and NBEAL1, both members of the BEACH domain-containing protein family, regulate CTLA-4 movement from Rab6-positive Golgi-derived tubules to the plasma membrane. Note: the length of ubiquitin chains is not depicted here. Ub, ubiquitin; MVB, multivesicular body; LRBA, LPS-responsive beige-like anchor protein; NBEAL1, neurobeachin-like protein 1; DEF6, differentially expressed in FDCP6 homolog.

CTLA-4 was the very first immune checkpoint receptor to be clinically targeted, marking the foundational breakthrough in the field of cancer immunotherapy [177,178]. In comparison with PD-1/PD-L1 inhibitors, CTLA-4 inhibitors are unfortunately less well tolerated by patients due to high toxicity and immune-related adverse events [179]. In distinction to PD-1, CTLA-4 lacks the canonical inhibitory signalling motifs in its cytoplasmic tail. Instead, its immunosuppressive activity is primarily mediated through high-affinity competitive ligand binding with co-stimulatory receptor CD28, and the ability to down-regulate their common ligands, CD80 and CD86, from APCs via trans-endocytosis (Figure 4) [26,180].

#### Biosynthesis

Unlike PD-1 and PD-L1, which predominantly localise at the plasma membrane, immunofluorescence images show that CTLA-4 is found mainly in endosomal compartments in both epithelial and activated T cells at steady state [8,121,181,182]. CTLA-4 has two N-linked glycosylation sites within its extracellular domain (Asn113 and Asn145), which, upon double mutation, shift CTLA-4 from a mature (~29 kDa) down to a non-glycosylated form (~22 kDa) [183]. A polymorphism (Thr17Ala) within the N-terminal signal peptide of CTLA-4 correlates with incomplete CTLA-4 glycosylation and processing at the ER, leading to lower cell surface expression and autoimmune disorders [183]. In T cells, CTLA-4 delivery to the cell surface relies on the activity of phospholipase D and GTPase Arf-1 [182]. A multimeric complex comprising Rab8, immune cell-specific T cell receptor-interacting molecule (TRIM) and linker for activation of X cells (LAX) is also involved in transporting CTLA-4 to the cell surface in vesicles derived from the TGN [184].

##### Intracellular dynamics

CTLA-4 is mainly viewed as a T cell-specific receptor, but independent studies indicate that it is also present in some cancer cells [8,121]. Pioneering work from the Sansom lab has revealed a general conservation of the CTLA-4 trafficking itinerary across T cells and epithelial cells [181,182,185–187]. CTLA-4 dynamics within the endo-lysosomal network are predominantly mediated by its short cytoplasmic domain. This region is conserved across mammals but varies considerably in primitive species [185]. Tailless CTLA-4 loses its characteristic intracellular distribution and redistributes to the plasma membrane instead [188,189]. The high endocytic activity of CTLA-4 is crucial to limiting ligand availability to co-stimulatory receptor CD28, as CTLA-4 can down-regulate their shared ligands via trans-endocytosis (Figure 4) [187,190,191]. During this process, CTLA-4 interacts with and internalises its ligands for lysosomal degradation, whilst CTLA-4 itself can have two distinct fates depending on its ligand interaction. CD86-bound CTLA-4 dissociates from the ligand in a pH-dependent manner and recycles back to the plasma membrane, whereas CD80-bound CTLA-4 is sorted to the lysosomal pathway for degradation [192]. This mode of action seems to be an inherent characteristic of CTLA-4 since its expression is sufficient to impart this function irrespective of the cell types in which it is expressed [187,190]. Trans-endocytosis of CD80 triggers CTLA-4 ubiquitylation, but the specific E3 ligases involved remain unidentified. CD86 or CTLA-4 ubiquitylation is not observed during trans-endocytosis of CD86, but in dendritic cells, CD86 is known to be ubiquitylated by MARCH1 for directing its internalisation and lysosomal degradation [161].

T-cell activation increases CTLA-4 surface presentation without completely stabilising it at the plasma membrane [91,181,193]. CTLA-4 internalisation into CCVs requires dynamin and its interaction with the clathrin adaptor AP-2 via its membrane-proximal endocytic YVKM motif [181,194–198]. CTLA-4 contains a second membrane-distal tyrosine-based YFIP motif, which provides an alternative AP-2 binding site that is less efficient in directing endocytosis [188,189]. Early work suggested that phosphorylation of the tyrosine residue within this YVKM motif interferes with CTLA-4 binding to AP-2 and prevents its endocytosis in activated T cells [195,196,198]. This is corroborated by biochemical assays and yeast two-hybrid experiments from independent groups showing that phosphorylated CTLA-4 fails to interact with AP-2 [195,196,198]. These studies rely on overexpression systems or a broad-spectrum tyrosine phosphatase inhibitor to assess if phosphorylation affects CTLA-4 internalisation in T cell systems [195,196,198]. However, subsequent studies revealed constitutive CTLA-4 internalisation throughout T-cell activation, with approximately 80% of surface CTLA-4 being endocytosed within 5 mins [181,182]. These latter studies undertake a near-physiological approach by visualising CTLA-4 using a fluorescently conjugated antibody at 37°C in live primary T cells post-stimulation [181]. The question of whether CTLA-4 undergoes phosphorylation during physiological T cell activation and its contribution to CTLA-4 dynamics remains a lively topic in the field [180].

Endocytosed CTLA-4 undergoes rapid lysosomal degradation that is dependent on its ubiquitylation at Lys203 and Lys213 within its cytoplasmic tail [121]. CTLA-4 internalisation and lysosomal degradation require Rab5 and Rab7, which are the key Rab GTPases that regulate endosome maturation [186,199]. Interestingly, CTLA-4 sorting into the endo-lysosome pathway is independent of HRS (ESCRT-0 component) but still requires an ESCRT-associated scaffold protein, His domain phosphotyrosine phosphatase (HD-PTP, a pseudo-phosphatase also known as PTPN23) [121,200]. HD-PTP recruits USP8 to deubiquitylate CTLA-4 in cancer cell lines, mouse CD4-positive T cells and cancer cell-derived exosomes. In the absence of USP8, CTLA-4 is stabilised despite an increase in its ubiquitylation due to the pleiotropic effects of USP8 loss on the endo-lysosomal pathways that block lysosomal CTLA-4 turnover [121]. In response to v-ATPase inhibition, a pool of CTLA-4 can be secreted from cancer cells via exosomes, presenting its ligand-binding domain to the extracellular milieu [121]. Whilst the biological activity of exosome-derived CTLA-4 has not been investigated, soluble CTLA-4, a CTLA-4 isoform lacking the transmembrane domain, is functionally active and capable of attenuating T cell responses [201].

On average, plasma membrane proteins are generally longer-lived (t_1/2_ ~100 h) than intracellular proteins (t_1/2_ ~60 h) in tissue culture cells [202]. With less than 5% of the human proteome being considered short-lived (t_1/2_ < 8 h), CTLA-4 stands out as a remarkably unstable membrane receptor across cancer cells and T cells (Table 2) [82,91,121]. This then raises the question: why is CTLA-4 so unstable? Through a ubiquitin chain restriction (UbiCRest) analysis aimed at decoding the linkage composition of CTLA-4, we recently identified a complex polyubiquitin chain architecture featuring Lys63-, Lys27- and Lys29-linked polyubiquitin chains on CTLA-4 [121]. The Lys63-linked ubiquitin chain is prominently associated with receptor endocytosis, but less is known about the involvement of Lys27 and Lys29 linkages in directing receptor sorting and degradation [62,75,203]. In principle, heterotypic polyubiquitin chains may serve as a superior lysosomal degradative signal over monoubiquitylation or homotypic chains, as multiple ubiquitin molecules and chain topologies may afford higher avidity for interaction between the ubiquitylated receptor and the ubiquitin-binding ESCRT components. This concept draws from similar findings that heterotypic branched ubiquitin chains have been shown to enhance cytosolic protein degradation by the proteasome [204,205]. TRAF6 is so far the only E3 ligase reported to ubiquitylate CTLA-4 in activated CD4-positive and CD8-positive murine T cells [160]. It is known to mediate Lys63-linked ubiquitylation on itself and its substrate, and it controls multiple immune signalling cascades via its enzymatic and scaffolding activities [206–209]. Currently, it remains unclear if other E3 ligases are implicated in the fast turnover of CTLA-4.

The retention using selective hook (RUSH) system is a two-component assay that uses an ER-anchored hook containing streptavidin to retain a protein of interest fused to a streptavidin-binding peptide [210]. The addition of biotin enables the synchronised release of the protein of interest from the hook for real-time tracking through the secretory pathway. A CTLA-4-RUSH assay showed that CTLA-4 follows the conventional biosynthetic route to reach the plasma membrane, followed by its endocytosis and recycling [211]. A key molecule that controls CTLA-4 surface presentation is LPS-responsive beige-like anchor protein (LRBA). LRBA belongs to the Beige and Chediak-Higashi (BEACH) domain family, which is a highly conserved family of large proteins involved in membrane trafficking [212]. It was first implicated in CTLA-4 trafficking following reports of LRBA-deficient patients who developed autoimmune disorders resembling CTLA-4 deficiency [22]. This phenotype is attributed to accelerated CTLA-4 degradation in the absence of LRBA, suggesting that LRBA normally protects CTLA-4 from lysosomal targeting. LRBA depletion also leads to CTLA-4 loss in murine regulatory T cells but without progression to immune dysregulation [213]. One study, nevertheless, reports normal CTLA-4 expression and regulatory T-cell function in an LRBA-deficient patient with abnormal immunity, suggesting that other cellular players may be involved in governing CTLA-4 surface presentation [214].

Since the first report linking LRBA to CTLA-4, we are only starting to grasp how it controls CTLA-4 trafficking and what other roles it may have beyond regulating CTLA-4. LRBA localises to both Golgi and endosomal compartments, with its recruitment to the endosomes dependent on the GTPases Arf1 and Arf3 [215]. It is essential for preserving endolysosomal homeostasis, as its deficiency leads to enlarged yet proteolytically active endolysosomes [215]. Two distinct endosomal recycling pathways have been defined: a fast route dependent on the small GTPase Rab4, and a slow route mediated by Rab11 [216,217]. LRBA was initially shown to mediate CTLA-4 recycling via Rab11-positive slow recycling endosomes [22,186]. However, a recent systematic analysis revealed that the majority of endosomal LRBA associates with Rab4-positive fast recycling endosomes, whilst the Golgi-associated LRBA co-localises with Rab6 [215]. The RUSH assay mentioned above further showed that CTLA-4 transits along the Golgi-derived tubules enriched for LRBA and neurobeachin-like protein 1 (NBEAL1, another BEACH domain-containing protein), and a small fraction of CTLA-4 reaches Rab11-positive endosomes directly from the Golgi [211]. These observations suggest that LRBA may co-ordinate CTLA-4 trafficking not just along the established endosomal recycling pathway but also through the biosynthetic route. A pulldown of wildtype or GST-tagged CTLA-4 Lys mutants with either purified BEACH domain-containing proteins or HeLa cell lysates showed that the main CTLA-4 ubiquitylation sites, Lys203 and Lys213, are essential for its interaction with this class of cargo-sorting adaptors [121,211]. LRBA appears to contain a ubiquitin-binding VHS domain, which is also present, most notably, in the ESCRT-0 protein HRS [212,218]. These findings suggest that unmodified Lys residues may be a prerequisite for CTLA-4 binding to LRBA and that ubiquitylation at these sites may destroy this interaction, potentially overriding LRBA-dependent recycling and redirecting CTLA-4 towards lysosomal degradation.

LRBA is proposed to deflect CTLA-4 from the lysosomal pathway by competing with the clathrin adaptor AP-1, since co-depletion of AP-1 and LRBA partially re-established the loss of CTLA-4 in LRBA-deficient cells [22]. An early study suggested that AP-1 delivers CTLA-4 from the TGN to lysosomes by interacting with the membrane-proximal YVKM motif of CTLA-4 [197]. This model is based on the observations that (i) CTLA-4 interacts with AP-1 and (ii) the CTLA-4 YVKM mutant is lost in the lysosomal and Golgi fractions in a subcellular fractionation experiment [197]. Considering the YVKM motif is known to direct AP-2 and clathrin-dependent CTLA-4 endocytosis, an alternative interpretation is that the endocytosis-incompetent CTLA-4 YVKM mutant is anchored at the plasma membrane and is therefore unable to be delivered to the lysosome or recycled back to the Golgi compartments. In line with this, tracking CTLA-4 movement from the ER using the RUSH system confirms that CTLA-4 is excluded from AP-1-coated vesicles [211]. AP-1 is known to retrieve the lysosomal hydrolase mannose-6-phosphate receptors (M6PRs) from the endosomes back to the TGN, and its deficiency compromises the delivery of lysosomal enzymes in the cells [219–222]. Thus, the rescue phenotype in LRBA- and AP-1 double-depleted cells is likely a consequence of the mis-sorting of lysosomal enzymes upon AP-1 depletion rather than a reduced CTLA4 Golgi-to-lysosome transport in the absence of AP-1.

Similar to LRBA deficiency, mutations in differentially expressed in FDCP6 homolog (DEF6) have also been shown to affect CTLA-4 trafficking in T cells [223]. DEF6 is a T cell-specific guanine exchange factor (GEF) for Rab11, and its deficiency is proposed to affect CTLA-4 recycling and surface abundance on T cells. CTLA-4 also interacts with the endosomal SNX-BAR sorting complex for promoting exit-1 (ESCPE-1) subunits SNX1/2/5/6 and the retromer subunit VPS35, but the functional implications of its interaction with these recycling complexes have yet to be established [224]. Whilst we are beginning to have a clearer picture of the CTLA-4 trafficking itinerary in cells (Figure 4), the molecular details balancing its recycling and degradation, following ligand internalisation, are less clear.

### LAG-3

**Figure 5:**
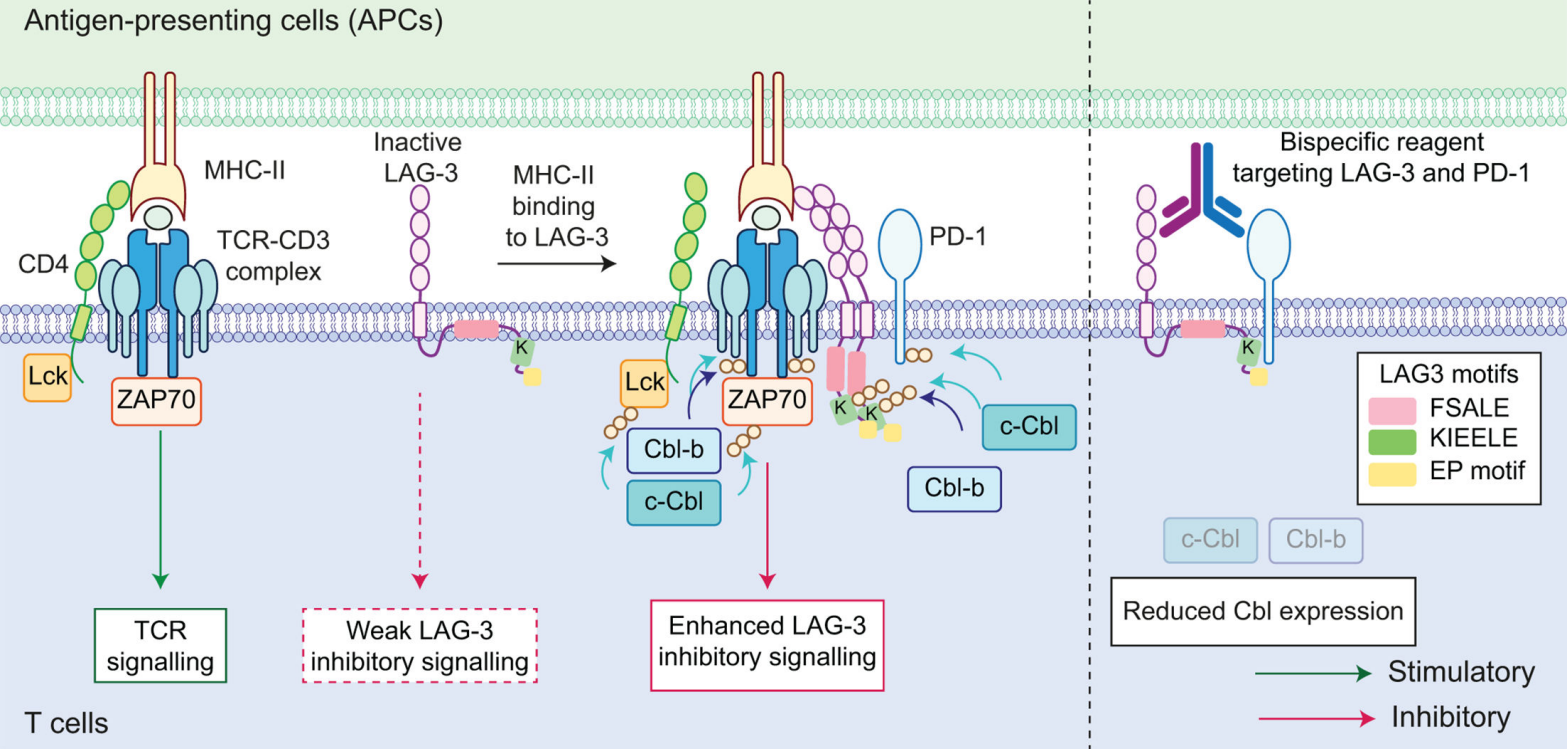
The Cbl E3 ligases lie at the heart of TCR signalling. The interaction between the TCR–CD3 complex and MHC-II is normally stabilised by CD4 in T cells. TCR activation triggers a signalling cascade that leads to the recruitment of the Cbl E3 ligases and translocation of LAG-3 and PD-1 to the TCR microcluster. Cbl E3 ligases ubiquitylate the TCR–CD3 complex and its associated signalling intermediates, leading to their down-regulation. Meanwhile, LAG-3 dimerisation and binding to MHC-II promote its ubiquitylation by the Cbl family, allowing the release of its cytoplasmic tail from the plasma membrane to reveal the hidden FSALE motif and promote strong LAG3 inhibitory signalling. Bispecific reagent targeting LAG-3 and PD-1 leads to a striking reduction in the Cbl ligases via unknown mechanisms, lifting the inhibitory signals and promoting antitumour effects.

The combination of antibodies directed against PD-1 (nivolumab) and LAG-3 (relatlimab) is reported to significantly enhance the progression-free survival of melanoma patients compared with PD-1 blockade alone [225,226]. This has garnered a lot of interest in the field, as PD-1 and LAG-3 have both been shown to accumulate at TCR microclusters [172,227]. LAG-3 dimerisation is critical for its inhibitory function. It does not have the classical ITIM or ITSM inhibitory motifs, but it contains three evolutionarily conserved motifs (a membrane-proximal FSALE motif, a KIEELE motif and a membrane-distal EP repeat) that are crucial for its immunosuppressive role (Figure 5). Whilst PD-1 suppresses late TCR signalling via SHP2-mediated dephosphorylation, LAG-3 appears to disrupt proximal TCR events. The engagement of the TCR–CD3 complex with MHC-II typically requires stabilisation by the co-receptor CD4 (Figure 5), which shares ~25% sequence identity with LAG-3 [228,229]. Current evidence suggests that LAG-3 may restrict the access of the TCR complex to its tyrosine kinase Lck and sterically hinder the stabilisation of the TCR–MHC-II interaction by CD4 through a non-overlapping mode of action [227,230,231]. Additionally, a recent study uncovered that MHC-II binding to LAG-3 triggers trans-endocytosis of MHC-II into T cells via a TCR-driven internalisation machinery to limit antigen presentation [232].

LAG-3 is distinct from the other immune checkpoint receptors as its shedding from the plasma membrane is key to controlling its bioavailability and inhibitory activity. Plasma membrane LAG-3 is cleaved by ADAM10 and ADAM17 into soluble and cytoplasmic fragments in a process that follows first-order kinetics (cleavage rate of ~5 h) [233,234]. Current evidence suggests that soluble LAG-3 may be inert. Given that the cytoplasmic tail of LAG-3 contains three conserved motifs essential for its inhibitory signal, it would be of interest to evaluate whether the cleaved cytoplasmic portion of LAG-3 stays functionally active.

The reported protein half-life of under 2 h suggests that there are pathways to LAG-3 degradation beyond membrane shedding (Table 2). In both unstimulated and stimulated T cells, perturbation of lysosomal acidification by concanamycin A prevents LAG-3 degradation [122]. This suggests an endo-lysosomal trafficking route for LAG-3, but molecular details are missing. At steady state, LAG-3 primarily localises in EEA1 (early endosome), Rab11 (recycling endosomes) and Rab27a (MVB) positive endosomes in activated T cells [235]. This large intracellular pool of LAG-3 is proposed to facilitate its rapid distribution to the plasma membrane following T-cell activation to suppress T-cell signalling [235].

The significance of ubiquitylation has recently been put in the spotlight in relation to LAG-3 signalling function rather than its degradation [123]. LAG-3 contains a single Lys (Lys498) residue within the KIEELE motif in its cytoplasmic tail. In the absence of a LAG3 ligand, the membrane-proximal FSALE motif is embedded within the plasma membrane, whereby LAG-3 mediates its weak inhibitory function mainly via its exposed EP motif (Figure 5) [123]. Immediately after MHC-II stimulation, about 4–7% of LAG-3 undergoes non-Lys48-linked ubiquitylation by the Cbl ligases, c-Cbl and Cbl-b [123]. Rather than promoting LAG-3 degradation, this ligand-induced Lys ubiquitylation activates and enhances LAG-3 inhibitory function by releasing the membrane-proximal FSALE signalling motif from the plasma membrane [123]. In accordance with the above, a LAG-3 mutant lacking this Lys residue still preserves its short-lived nature despite a complete loss of its ubiquitylation [123].

During TCR signalling, c-Cbl and Cbl-b are brought into proximity of the TCR–CD3 complex either directly via their phosphotyrosine-binding (PTB) domains or indirectly by binding to SH2/SH3 adaptor proteins such as Crk [236–240]. Upon recruitment, Cbl is phosphorylated by Src-family kinases downstream of TCR signalling, which enhances its E3 ubiquitin ligase activity to drive the ubiquitylation and degradation of the TCR–CD3 complex and associated kinases like ZAP70 and Lck (Figure 5) [241–243]. Available datasets for phosphorylation give no indication of phospho-tyrosine modification in the LAG-3 cytoplasmic domain [244]. Thus, we propose a model whereby recruitment of the Cbl E3 ligases during TCR signalling may down-regulate this signalling complex and simultaneously activate the counterbalance provided by LAG3 inhibitory signalling via ubiquitylation (Figure 5). Interestingly, PD-1, which co-localises with LAG-3 at the TCR microclusters, is also a target of c-Cbl [108,123,245]. A bispecific antibody reagent targeting both PD-1 and LAG-3 leads to stark down-regulation of c-Cbl in T cells isolated from non-small cell lung cancer patients, whilst antibodies against each receptor or even in combination have minimal effect [226]. Meanwhile, Cbl-b in T cells from these patients is also down-regulated by this reagent and antibodies against each receptor [226]. Thus, a complex system of checks and balances involving Cbl E3 ligases lies at the heart of T cell activation.

Serine, threonine and cysteine on the substrate proteins can also accept ubiquitin via the more labile hydroxyester (serine and threonine) or thioester (cysteine) linkages [246,247]. The presence of ester-linked ubiquitin can generally be confirmed by assessing the sensitivity of immunoprecipitated proteins to hydroxylamine treatment, which selectively cleaves the ester bonds. The short half-life of Lys-less LAG-3 mutant may represent a rare Lys-independent mode of receptor turnover rather than a ubiquitin-independent mode of degradation. This is further backed by the identification of the two conserved serine residues (Ser484 and Ser497) in the cytoplasmic tail of LAG-3 that are targeted for ubiquitylation by the linear ubiquitin chain assembly complex (LUBAC), at least in an epithelial cell culture line [162]. LUBAC is a ternary complex composed of the accessory protein, Shank-associated RH domain-interacting protein (SHARPIN), and two ubiquitin E3 ligases – heme-oxidised IRP2 ubiquitin ligase 1 (HOIL-1) and HOIL-1-interacting protein (HOIP). HOIL-1 is known to form ester linkages between the substrate and ubiquitin, whilst HOIP polymerises linear (M1-linked) chains [248,249]. Whether HOIL-1 catalyses the ester linkage on LAG-3 remains unclear, but the HOIP subunit of LUBAC is shown to generate linear ubiquitin chains on LAG-3, which can be cleaved by an M1-specific DUB, OTULIN [162]. As mentioned above, MHC-II binding triggers the generation of non-Lys48-linked chains on LAG3, but whether it generates M1 chains and if OTULIN removes this signal from ubiquitylated LAG-3 in stimulated T cells remains elusive [123].

### TIM-3

TIM-3 has long been debated to be either an immune checkpoint or a stimulatory receptor, mainly due to its diverse set of ligands, lack of a clearly defined inhibitory signalling motif and the reliance on the phosphorylation of two highly conserved tyrosine (Tyr256 and Tyr263) residues to mediate its signalling function, which can be associated with either stimulatory or inhibitory effects [18]. One of the strong pieces of evidence implicating TIM-3 as an immune checkpoint receptor came from independent studies of germline loss-of-function mutations (Tyr82Cys or Ile97Met) of *HAVCR2* (encoding TIM-3) in non-Hodgkin lymphoma patients. Patients harbouring these mutations suffer severe autoinflammatory diseases due to impaired TIM-3 folding that down-regulates its surface expression [23,250]. TIM-3 mutants aggregate in the Golgi and lose their plasma membrane presentation in myeloid and T cells [23,250]. These mutations are suggested to hinder TIM-3 glycosylation, but the underlying mechanisms remain elusive [23]. Another homozygous TIM-3 mutation (Thr101Ile), found in patients with inflammatory bowel disease, reportedly prevents TIM-3 glycosylation and trafficking to the plasma membrane [251].

Palmitoylation of TIM-3 at Cys296 by palmitoyltransferase DHHC9 in the ER facilitates its effective delivery to the cell surface [164]. When palmitoylation is blocked, prolonged TIM-3 residence in the ER leads to its ubiquitylation by HRD1 ligase for proteasomal clearance via the ERAD pathway [164]. Similar to LAG-3, TIM-3 is shed from the cell surface by ADAM10 and ADAM17, but the biological function of soluble TIM-3 is not currently known [252]. Beyond its trafficking along the biosynthetic pathway and shedding from the plasma membrane, we currently lack the full view of how TIM-3 is dynamically maintained by the endocytic and recycling routes.

## Targeted protein degradation: an emerging modality in immune checkpoint therapy

The potential of immune checkpoint inhibitors is often restricted by low efficacy, drug resistance and immune-related adverse reactions [179]. Immune checkpoint blockades are classified as occupancy-driven pharmacology because their effectiveness ultimately relies on receptor presentation on the cell surface and persistent drug exposure [253]. This also means the current inhibitor-based regimens are unable to reach the immune checkpoint receptors located inside the cells.

One way of targeting these ‘intractable’ intracellular membrane proteins is by leveraging the ubiquitin-proteasome system to induce protein degradation via the so-called ‘targeted protein degradation (TPD)’ platform. The effect of TPD emulates protein depletion via genetic engineering (e.g. CRISPR or RNA interference), but without the need for genomic alteration. TPD is based on bifunctional molecules that hook up a target protein to a requisite cellular component (e.g. E3 ligase) to selectively modulate the ubiquitylation and degradation of target proteins [253,254]. Once a target protein is degraded, the activity of the degrader is no longer needed until the target protein is made again. This allows the TPD molecules to act through iterative cycles of activity, often at very low dosages [253]. Thus, TPD offers a superior and more potent approach with reduced side effects than conventional therapy. The first compound of this class, termed proteolysis targeting chimaeras (PROTACs), was originally proven effective at targeting membrane receptors (e.g. RTKs), and the approach has now been extended to degrading scaffold proteins and oncogenic enzymes that are otherwise hard to target by inhibitors alone [255–258].

Building on the advances in the PROTAC field, similar degrader-based technologies have begun to surface within the immuno-oncology therapeutic space. Among these are antibody-based PROTACs (AbTACs) and proteolysis-targeting antibodies (PROTABs) [259,260]. The plasma membrane and endomembrane represent physical barriers within the cells, such that the extracellular domain of membrane proteins, positioned towards the external environment or inside the endosomal lumen, is out of reach of the E3s and other cellular components. Both AbTACs and PROTABs operate on the same fundamental concept, that is, to use bispecific antibodies to recruit plasma membrane-associated E3 ligases, such as RNF43 and ZNRF3, to surface receptors like PD-L1 [259,260]. This induced proximity allows the cytosolic catalytic domains of these membrane-bound E3s to act on the cytoplasmic tail of PD-L1 for ubiquitylation and degradation. Membrane-penetrating PROTACs also offer an alternative approach to degrade membrane proteins. Based on the knowledge that PD-L1 palmitoylation by DHHC3 blocks its lysosomal degradation, a peptide-PROTAC has been developed to induce PD-L1 degradation indirectly by degrading its cellular regulator, DHHC3, that would otherwise promote PD-L1 recycling [129,261]. Another PD-L1 degrader peptide, termed PD-LYSO, combines a PD-L1 binding sequence from HIP1R with a lysosomal sorting motif and has also been shown to deplete cellular PD-L1 levels [113].

An alternative facet of TPD exploits the trafficking kinetics of well-studied shuttling receptors to target membrane receptors and extracellular proteins for degradation. This concept was first introduced through the lysosome-targeting chimaeras (LYTACs) and has since been expanded to include tumour immune cell targeting chimaeras (TICTACs) and cytokine receptor-targeting chimaeras (KineTACs) [262–264]. LYTACs are synthetic molecules that simultaneously bridge the extracellular domains of the target receptor and a cell surface lysosome-shuttling receptor, such as the cation-independent mannose-6-phosphate receptor (CI-M6PR) [262,263]. This strategy relies on the unique biology of CI-M6PR that binds proteins modified with mannose-6-phosphate residues to mediate their endocytosis and lysosomal degradation, whilst CI-M6PR itself is recycled back to the plasma membrane [265]. A LYTAC composed of an FDA-approved antibody atezolimumab (for PD-L1 binding) and a glycopeptide ligand (for CI-M6PR binding) accelerates PD-L1 lysosomal degradation, resulting in a near 70% loss of PD-L1 in tissue culture cells [262]. LYTAC has recently been combined with bio-orthogonal endocytosis-targeting proteins, or EndoTags in short, to create fully proteinaceous protein-LYTAC (pLYTAC) [266]. EndoTags use *de novo*-designed proteins to bind to and trigger endocytosis of target proteins [266]. pLYTAC effectively degrades more than 80% of PD-L1 in tissue culture cells and mouse tumour models, improving the survival of treated mice compared with those receiving anti-PD-L1 [266]. Another new addition to the degrader-based technology targeting the immune checkpoint is the small molecule VISTA inhibitor-degrader [267]. It binds to VISTA with high affinity and promotes its degradation via the autophagy pathway [267].

## Concluding remarks

The first step towards proteostasis-based therapy in immune-oncology begins with a deep understanding of the fundamental cell biology of immune checkpoint receptors. Our current knowledge base in this field is, unfortunately, limited by some inconsistencies and a lack of reproducibility. The most consistent body of data relating to ubiquitin-modifying enzymes highlights key roles of the RING E3 ligase, Cbl, which has long been recognised as a negative regulator of T cell signalling [268]. Mice deficient in c-Cbl displayed thymic developmental defects due to a dysregulated signalling threshold during thymic selection, whilst Cbl-b knockout mice succumbed to spontaneous autoimmune disorders driven by hyperactive peripheral T cells [269,270]. A more far-reaching role in immune regulation has been proposed in both stimulatory and inhibitory pathways. Cbl family proteins have emerged as common regulators of PD-1 and LAG-3, whilst co-inhibition of both receptors in turn can regulate Cbl expression [226]. We look forward to the further elaboration of ubiquitin-dependent pathways relating to checkpoint receptors and suggest that this emerging field is ripe for exploration.

## Abbreviations

APCs: antigen-presenting cells
AbTACs: antibody-based PROTACs
BEACH: Beige and Chediak-Higashi
CADM1: cell adhesion molecule 1
CCVs: clathrin-coated vesicles
CEACAM1: Carcinoembryonic antigen-related cell adhesion molecule 1
CMTM6: CKLF-like MARVEL transmembrane domain containing 6
CORVET: class C core vacuole/endosome tethering
CTLA-4: Cytotoxic T lymphocyte associated antigen 4
DEF6: differentially expressed in FDCP6 homolog
DUBs: deubiquitylases
ERAD: endoplasmic reticulum-associated protein degradation
ESCPE-1: endosomal SNX-BAR sorting complex for promoting exit-1
ESCRT: endosomal sorting complex required for transport
FACS: Fluorescence-Activated Cell Sorting
FGL1: fibrinogen-like protein 1
GEF: guanine exchange factor
GFP: Green fluorescent protein
GGT5: glutathione hydrolase 5 proenzyme
GRAIL: Gene related to anergy in lymphocytes
HC: heavy chain
HCMV: human cytomegalovirus
HD-PTP: His domain phosphotyrosine phosphatase
HIP1R: Huntingtin-interacting protein 1
HMGB1: high mobility group protein B1
HOIL-1: heme-oxidised IRP2 ubiquitin ligase 1
HOIP: HOIL-1-interacting protein
HOPS: homotypic fusion and vacuolar protein sorting
ILVs: intraluminal vesicles
ITIM: immunoreceptor tyrosine-based inhibitory motif
KSHV: Kaposi’s sarcoma-associated herpesvirus
KineTACs: cytokine receptor-targeting chimaeras
LAG-3: Lymphocyte-Activation Gene 3
LAX: linker for activation of X cells
LRBA: LPS-responsive beige-like anchor
LUBAC: linear ubiquitin chain assembly complex
LYTACs: lysosome-targeting chimaeras
Lys: lysine
MHC: major histocompatibility complex
M6PRs: mannose-6-phosphate receptors
MVBs: multivesicular bodies
Met: methionine
NBEAL1: neurobeachin-like protein 1
PD-1: programmed cell death protein 1
PD-L1: programmed death ligand 1
PD-L2: programmed death ligand 2
PLD: phospholipase D
PROTABs: proteolysis-targeting antibodies
PROTACs: proteolysis targeting chimaeras
PTB: phosphotyrosine-binding
RING: really interesting new gene
RTKs: receptor tyrosine kinase
RUSH: retention using selective hook
SCARF2: scavenger receptor class F member 2
SHARPIN: Shank-associated RH domain-interacting protein
STXBP6: syntaxin binding protein 6
TCR: T cell receptor
TGN: trans-Golgi network
TICTACs: tumour immune cell targeting chimaeras
TIGIT: T cell immunoreceptor with immunoglobulin and ITIM domains
TIM-3: T cell immunoglobulin and mucin domain-containing protein 3
TMUB1: transmembrane and ubiquitin-like domain-containing protein 1
TOX: thymocyte selection-associated high mobility group box protein
TPD: targeted protein degradation
TRAPPC4: Trafficking protein particle complex subunit 4
TRIM: T cell receptor-interacting molecule
VISTA: V-domain Ig suppressor of T cell activation
VSIG-3: V-Set and Immunoglobulin domain containing 3
pLYTAC: protein-LYTAC
β2m: β2-microglobulin
